# Evaluation of the accuracy of diagnostic coding for influenza compared to laboratory results: the availability of test results before hospital discharge facilitates improved coding accuracy

**DOI:** 10.1186/s12911-021-01531-9

**Published:** 2021-05-22

**Authors:** Nasir Wabe, Ling Li, Robert Lindeman, Jeffrey J. Post, Maria R. Dahm, Julie Li, Johanna I. Westbrook, Andrew Georgiou

**Affiliations:** 1 Centre for Health Systems and Safety Research, Australian Institute of Health Innovation, Macquarie University, North Ryde, NSW 2109 Australia; 2 New South Wales Health Pathology, St Leonards, NSW 2065 Australia; 3 Department of Infectious Diseases, Prince of Wales Hospital, Randwick, NSW 2031 Australia; 4 Prince of Wales Clinical School, University of New South Wales, Kensington, NSW 2052 Australia; 5 Institute for Communication in Health Care, The Australian National University, 110 Ellery Crescent, Acton, ACT 2601 Australia

**Keywords:** ICD-10-AM, Diagnostic codes, Influenza diagnosis, Diagnostic accuracy

## Abstract

**Background:**

Assessing the accuracy of diagnostic coding is essential to ensure the validity and reliability of administrative coded data. The aim of the study was to evaluate the accuracy of assigned International Classification of Diseases version 10-Australian Modification (ICD-10-AM) codes for influenza by comparing with patients’ results of their polymerase chain reaction (PCR)-based laboratory tests.

**Method:**

A retrospective study was conducted across seven public hospitals in New South Wales, Australia. A total of 16,439 patients who were admitted and tested by either cartridge-based rapid PCR or batched multiplex PCR between January 2016 and December 2017 met the inclusion criteria. We calculated the sensitivity, specificity, positive predictive value (PPV) and negative predictive value (NPV) of ICD-10-AM coding using laboratory results as a *gold standard*. Separate analyses were conducted to determine whether the availability of test results at the time of hospital discharge influenced diagnostic coding accuracy.

**Results:**

Laboratory results revealed 2759 positive influenza cases, while ICD-10-AM coding identified 2527 patients. Overall, 13.7% (n = 378) of test positive patients were not assigned an ICD-10-AM code for influenza. A further 5.8% (n = 146) patients with negative test results were incorrectly assigned an ICD-10-AM code for influenza. The sensitivity, specificity, PPV and NPV of ICD-10-AM coding were 93.1%; 98.9%; 94.5% and 98.6% respectively when test results were received before discharge and 32.7%; 99.2%; 87.8% and 89.8% respectively when test results were not available at discharge. The sensitivity of ICD-10-AM coding varied significantly across hospitals. The use of rapid PCR or hospitalisation during the influenza season were associated with greater coding accuracy.

**Conclusion:**

Although ICD-10-AM coding for influenza demonstrated high accuracy when laboratory results were received before discharge, its sensitivity was substantially lower for patients whose test results were not available at discharge. The timely availability of laboratory test results during the episode of care could contribute to improved coding accuracy.

**Supplementary Information:**

The online version contains supplementary material available at 10.1186/s12911-021-01531-9.

## 1. Introduction

Influenza is an acute respiratory infection caused by influenza A or B viruses. Seasonal influenza continues to be a major public health concern causing significant morbidity and mortality globally [1, 2]. Whilst the diagnosis of influenza can be made clinically based on presenting signs and symptoms, laboratory testing is recommended to confirm the infection, especially for patients hospitalised with acute respiratory illness or at high risk of complications [3]. There are a number of laboratory tests available to diagnose influenza including serological techniques such as enzyme immunoassay and complement fixation, viral culture and antigen-based tests. However, molecular methods based on reverse transcription polymerase chain reaction (PCR) are preferred because of their ability to detect multiple respiratory viruses simultaneously with high sensitivity and specificity [4, 5] and their potential to improve clinical management and workflow [6–8].

The International Classification of Diseases (ICD) is an integral part of the process for monitoring disease prevalence in Australia and internationally. It is a universally accepted standard diagnostic coding system used to classify diseases and other health problems for clinical, research and health management purposes. It is also used for supporting decision-making on reimbursement and resource allocation, surveillance and public reporting, and provision of mortality and morbidity statistics [9]. In Australia, ICD version 10 with Australian Modification (ICD-10-AM) was introduced in 1998 in hospitals and other healthcare agencies [10]. ICD-10-AM codes are assigned by trained clinical coders based on information in the medical record and the data are stored in an administrative database.

Assessing the accuracy of diagnostic coding is essential to ensure the validity and reliability of administrative diagnostic data and its usefulness for the intended purposes. Previous studies have utilised different approaches to assess the accuracy of ICD codes by comparing with other diagnostic methods [11, 12] and laboratory results [13–19] or by conducting independent manual reviews of hospital case notes or discharge summaries [20–23]. Given that the microbiological detection of a disease-causing organism using highly sensitive and specific laboratory tests such as culture and PCR-based tests are considered the *gold standard* in the diagnosis of many infection diseases [24], the use of laboratory results as a reference standard can be considered an ideal approach to evaluate accuracy of assigned ICD codes. Previous studies have reported a low to moderate level of coding accuracy compared with laboratory diagnoses for other infectious diseases [13–19, 21]. Studies that have assessed the accuracy of diagnostic coding for influenza [17, 18] have been limited to paediatric populations using the ICD-9 coding system.

In this study we used routinely collected administrative data from seven Australian hospitals over two years (2016–2017) to evaluate the accuracy of ICD-10-AM coding for influenza compared with results of PCR-based laboratory tests. In addition, we assessed whether the availability of laboratory results before hospital discharge improved coding accuracy. We hypothesized that patients whose laboratory results were received before discharge are more likely to have accurate coding compared to those whose laboratory results were received after discharge.

## Methods

### Study design and setting

A retrospective observational study was conducted across seven public hospitals in New South Wales, Australia. Hospitals A, B, C and G are located in metropolitan Sydney and Hospitals D to F are located within another Local Health District. Hospitals A to F are general hospitals while Hospital G is a children’s hospital. Each hospital offers a comprehensive range of inpatient and community services. In 2016–17 [25], the hospitals had total admissions of 65,793 (Hospital A), 48,151 (Hospital B), 28,772 (Hospital C), 51,659 (Hospital D), 16,603 (Hospital E), 21,266 (Hospital F) and 18,787 (Hospital G). All seven hospitals were served by a single pathology laboratory provider.

### Participants and data sources

The study period was between 1 January 2016 and 31 December 2017. All consecutive patients who were ordered laboratory tests while in the hospital to detect influenza (multiplex or rapid PCR) with or without a diagnosis of influenza (ICD-10-AM J09-J11) during the study period were included in the study. Given that the purpose of the study was to evaluate influenza diagnosis against laboratory findings, patients who were recorded by ICD-10-AM as having influenza but who were not ordered a laboratory test to detect influenza were excluded from the study. Moreover, patients who had the PCR tests after hospital discharge and who did not have ICD10 coding were not included in the current study.

Laboratory test data were obtained by linking the Laboratory Information System (LIS) and Admitted Patient Data Collection (APDC). Data linkage was achieved using de-identified patient medical records, gender, hospital, date of birth and datetimes of laboratory tests and hospital admissions in a similar approach as previously described [26, 27]. Relevant laboratory-related data obtained from the LIS included the type of influenza test, test results, the settings where the test was ordered, and the time a specimen was received at the laboratory and a verified result was available. Two types of tests were available for the detection of influenza during the study period: the batched multiplex PCR (multiplex PCR *hereafter*) and the newly introduced, rapid PCR. The characteristics of these tests, including the impacts on various ED and inpatient outcomes have been published elsewhere [8, 28]. Briefly, the multiplex PCR technology was the Seegene Allplex™ RP1/2/3 (Seoul, Republic of Korea) which can detect up to sixteen respiratory viruses including influenza A and B [29]. It was a referral test available at a central laboratory based at Hospital B, with a lengthy test turnaround time[8, 28]. The rapid PCR was a Cepheid’s Xpert® Flu/RSV XC (Sunnyvale, CA) performed at hospital-based laboratories resulting in shorter test turnaround time. Rapid PCR has demonstrated a high sensitivity and specificity for the rapid detection of influenza A/B and respiratory syncytial virus (RSV) [4].

The APDC contained information about inpatient details including principal and secondary diagnoses, admission and disposition times, source of referral, mode of separation and hospital length of stay. The updated version of the Charlson comorbidity index was calculated based on the ICD-10-AM codes[30]. The ICD-10-AM codes J09-J11 from the primary or secondary diagnoses were used to identify influenza diagnoses (Additional file 1: Table 1).

**Table 1:** Demographic and clinical characteristics of study population

Variable	Positive test results (n = 2759)	All patients (n = 16,439)
Female, n (%)	1414 (51.3)	7962 (48.4)
Age (years), median (IQR)	76 (57–85)	66 (18–81)
Season, n (%)		
Influenza^a^	2352 (85.3)	9016 (54.9)
Non-influenza	407 (14.7)	7423 (45.1)
Year of admission, n (%)		
2016	875 (31.7)	6487 (39.5)
2017	1884 (68.3)	9952 (60.5)
Setting where the test was ordered		
ED	1668 (60.5)	7750 (47.2)
Inpatient	994 (36.0)	8159 (49.6)
ED and inpatient	97 (3.5)	530 (3.2)
Source of referral, n (%)		
Emergency department	2511 (91.0)	13,890 (84.5)
Other^b^	248 (9.0)	2549 (15.5)
Mode of separation, n (%)		
Discharged by hospital	2215 (80.3)	13,454 (81.9)
Transferred to another setting	408 (14.8)	2119 (12.9)
Died in the hospital	100 (3.6)	596 (3.6)
Other (e.g. left at own risk)	36 (1.3)	270 (1.6)
Hospital, n (%)		
A	796 (28.9)	4145 (25.2)
B	566 (20.5)	2731 (16.6)
C	383 (13.9)	2051 (12.5)
D	493 (17.8)	2978 (18.1)
E	224 (8.1)	959 (5.8)
F	126 (4.6)	710 (4.3)
G	171 (6.2)	2865 (17.4)
Hospital length of stay (days), median (IQR)	4.7 (2.4–10.1)	5.1 (2.7–10.7)
Charlson comorbidity index, median (IQR)	1 (0–2)	1 (0–2)
ICD-10-AM principal diagnosis of influenza, n (%)	1281 (46.4)	1337 (8.1)

^a^July–October

^b^E.g. medical practitioner other than private, other hospital/day procedure centre and outpatients, community health

### Measures of diagnostic coding accuracy

The accuracy of ICD-10-AM coding for influenza [against PCR-based laboratory results as a *gold standard*] was evaluated using standard diagnostic accuracy measures comprising sensitivity, specificity, positive predictive value (PPV) and negative predictive value (NPV). Sensitivity was calculated as the proportion of test positive (PCR+) cases that were correctly recorded as ICD-10-AM J09-J11 (ICD10 +) –i.e. the number of PCR+/ ICD10+ cases divided by total PCR+ cases. Specificity was calculated as the proportion of test negative (PCR−) cases that were correctly identified by ICD-10-AM as such (ICD10−) –i.e. the number of PCR−/ICD10−  cases divided by total PCR− cases. PPV was calculated by dividing PCR+/ICD10+ cases by total ICD10+ cases and NPV was calculated by dividing PCR−/ ICD10− cases divided by total ICD10− cases.

Patients who were PCR+ but were not recorded as ICD-10-AM J09-J11 (ICD10−) were regarded as a *missed diagnosis*. The proportion of patients with *missed diagnoses* was calculated by dividing PCR+/ICD10− cases by total PCR+ cases. Patients who were PCR− but wrongly recorded as ICD10+ were regarded as a *miscoded diagnosis*. The proportion of patients with *miscoded diagnoses* were calculated in two ways: (i) by dividing PCR−/ICD10+ cases by total ICD10+ cases and (ii) by dividing PCR−/ICD10+ cases by total PCR− cases.

### Statistical analysis

Descriptive statistics including median with inter-quartile range (IQR) for continuous variables and percentages with 95% confidence interval for categorical data were reported where appropriate. A two-by-two table comparing influenza diagnosis status (ICD-10-AM J09-J11, + /−) and results of a laboratory test for influenza (PCR, +/−) was prepared for the overall sample and separately for patients whose laboratory results were received before or after hospital discharge. Binary logistic regression was used to determine the effect of laboratory result availability (i.e. before vs after discharge) on the likelihood of having correct ICD-10-AM coding when test result was positive (i.e. PCR+/ICD10+ after adjustment for potential confounders.

We conducted subgroup analyses by type of laboratory test (rapid vs multiplex PCR), setting where the test was ordered [emergency department (ED) vs inpatient], study hospital and season [influenza (July–October) vs non-influenza] to determine whether the sensitivity of ICD-10-AM coding differed by these variables. Given a significant interaction effect by laboratory result availability, separate data were presented for each group. The difference in the likelihood of having correct ICD-10-AM coding within each of these variables was assessed using a binary logistic regression. All analyses were adjusted for relevant demographic and clinical characteristics. The strength of the associations was measured using odds ratio (OR) with a 95% CI. P-values were 2-tailed and P < 0.05 was considered statistically significant. Analyses were conducted using Stata version 15 (StataCorp LP, College Station, TX).

## Results

### Participants

**Fig. 1:**
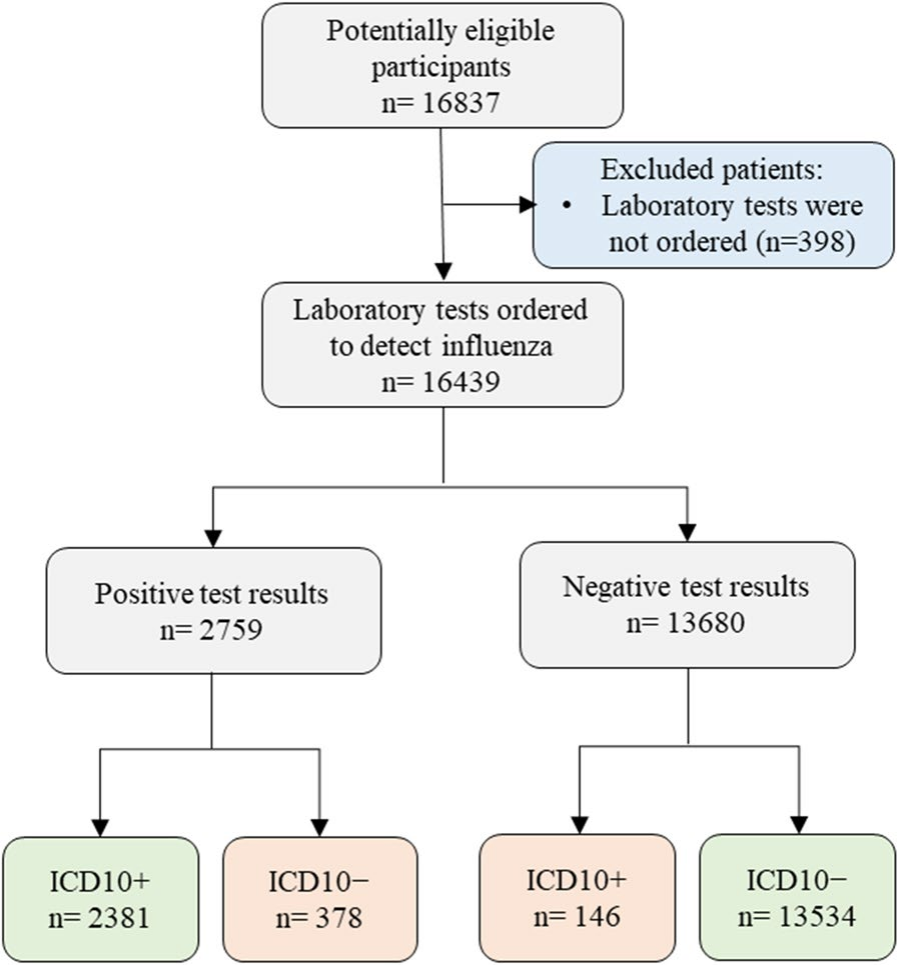
Patient selection flow chart. ICD10+ represents patients with and ICD10+ code for influenza; ICD10− no ICD10 code for influenza. Potentially eligible participants were patients who were ordered a laboratory test for influenza or were assigned ICD-10-AM codes for influenza

A total of 16,439 patients met the inclusion criteria (Fig. 1). Of these, 56.0% (n = 9214) were ordered multiplex PCR, 40.7% (n = 6690) rapid PCR and 3.3% (n = 535) were ordered both multiplex and rapid PCR. The median turnaround time was 3.6 h (IQR, 2.2–7.3) for rapid PCR and 26.6 h (IQR, 20.8–31.6) for multiplex PCR. Laboratory test results revealed that 16.8% (n = 2759) of patients were positive for either influenza A/B (influenza A only, n = 2,120; influenza B only, n = 632 and both influenza A and B, n = 7).

Overall, 2527 patients were assigned ICD-10-AM codes for influenza, of which 52.9% (n = 1337) recorded influenza as a primary diagnosis. The specific ICD-10-AM codes assigned are presented in the Additional file 1. J10.1 (i.e. *influenza with other respiratory manifestations, seasonal influenza virus identified)* was the most common diagnosis accounting for roughly two-thirds (61.6%) of all ICD-10-AM codes for influenza.

### Baseline demographic and clinical characteristics

The median age was 76 years for patients with positive test results and 66 years for the entire sample. Overall, the majority of patients (n = 13,890) were admitted through ED and 3.6% (n = 596) of patients died in hospital. The median hospital length of stay was approximately 5 days and patients had a median comorbidity index of one (Table 1).

### Accuracy of ICD-10-AM coding

A two-by-two table comparing the influenza diagnosis status vs laboratory test results is presented in Table 2. Of the 2759 patients who were PCR + , 2381 were assigned an ICD10+ influenza code. The proportion of patients with *missed* was 13.7% (n = 378). About 146 patients had a *miscoded diagnosis* [5.8% of all ICD10+ cases (146/2527) or 1.1% of all PCR– (146/13,680)]. Of patients with a *miscoded diagnosis*, 56 (38.4%) were recorded as primary and 90 (61.6%) as secondary diagnoses.

**Table 2:** Influenza diagnosis status versus laboratory test results

Influenza diagnosis	Laboratory results		
	PCR+	PCR−	Total
ICD10+	2381	146	2527
ICD10−	378	13,534	13,912
Total	2759	13,680	16,439
Accuracy measures (95% CI)			
Sensitivity	86.3 (85.0–87.6)		
Specificity	98.9 (98.7–99.1)		
PPV	94.3 (93.2–95.1)		
NPV	97.3 (97.0–97.5)		

*PPV* positive predictive value, *NPV* negative predictive value

The sensitivity, specificity, PPV and NPV of ICD-10-AM coding for influenza were 86.3, 98.9, 94.3 and 97.3% respectively (Table 2). The sensitivity varied considerably across hospitals from 63.7% at Hospital G to 99.2% at Hospital F, but specificity was high for all hospitals with values of > 97% across sites (Additional file 2).

Analysis by the type of influenza diagnosis (i.e. primary or secondary) revealed that the PPV of ICD-10-AM coding was higher for patients who had influenza as a primary diagnosis (PPV, 95.8; 95% CI, 94.6–96.8) compared with patients who had influenza as a secondary diagnosis (PPV, 92.4; 95% CI, 90.8–93.9).

### The effect of test result availability on coding accuracy

The majority of patients received test results before discharge (86.9%, n = 14,285) and positivity rate was 17.2% (n = 2450). Of patients whose test results were pending at discharge (n = 2154), 14.3% (n = 309) eventually came back positive for influenza. The sensitivity of ICD-10-AM coding for influenza was 93.1% for patients whose test results were received before discharge and 32.7% for patients whose test results were received after discharge: a statistically significant difference of 60.4% (*P* < 0.01). The specificity was very high for both groups (Table 3).

**Table 3:** Influenza diagnosis (ICD-10-AM J09-J11) versus laboratory test results by time of test result availability

Influenza diagnosis	Test results received before discharge (n = 14,285)			Test results after discharge (n = 2154)		
	PCR+	PCR−	Total	PCR+	PCR−	Total
ICD10+	2280	132	2412	101	14	115
ICD10−	170	11,703	11,873	208	1831	2039
Total	2450	11,835	14,285	309	1845	2154
Accuracy measures						
Sensitivity (95% CI)	93.1 (92.0–94.0)			32.7 (27.5–38.2)		
Specificity (95% CI)	98.9 (98.7–99.1)			99.2 (98.7–99.6)		
PPV (95% CI)	94.5 (93.5–95.4)			87.8 (80.4–93.2)		
NPV (95% CI)	98.6 (98.3–98.8)			89.8 (88.4–91.1)		

*PPV* positive predictive value, *NPV* negative predictive value

After adjusting for relevant confounders including hospital length of stay, patient age, comorbidity index, season, year of admission, hospital, setting where the test was ordered and type of laboratory test, patients whose test results were received before discharge was approximately 15 times more likely to have correct ICD-10-AM coding when test results were positive compared to patients whose results were received after discharge (OR, 15.2; 95% CI, 11.0–20.9).

### Subgroup analyses

Table 4 presents subgroup analyses by selected characteristics separately for patients whose test results were received before (n = 2450) or after discharge (n = 309) among patients who had positive results. Analysis by the type of laboratory test for influenza revealed that for patients whose test results were received before discharge, the sensitivity was significantly higher when rapid PCR was used compared to when multiplex PCR was used (96.6 vs 87.0%, *P* = 0.01) and the likelihood of having correct ICD-10-AM coding was two times higher for rapid PCR users (vs multiplex PCR users) (OR, 2.1; 95% CI, 1.3–3.5) after adjustment for potential confounders. For patients whose test results were received after discharge, the sensitivity was very low (< 33%) regardless of the type of laboratory test and there was no significant difference between patients who were ordered rapid or standard PCR (*P* = 0.46).

**Table 4:** The accuracy of influenza coding (ICD10 +) among patients with PCR + : A sub-group analysis by selected characteristics and test result availability

	Test results received before discharge (n = 2450)				Test results received after discharge (n = 309)			
	PCR+	ICD10+	Sensitivity (95% CI)	Adjusted OR^a^ (95% CI); P-value	PCR+	ICD10+	Sensitivity (95% CI)	Adjusted OR^a^ (95% CI); P-value
Type of laboratory test								
Rapid PCR^b^	1551	1498	96.6 (95.6–97.4)	2.1 (1.3–3.5); *P* = 0.01	20	6	30.0 (11.9–54.3)	1.6 (0.4–6.4); *P* = 0.46
Multiplex PCR	899	782	87.0 (84.6–89.1)	Ref	289	95	32.9 (27.5–38.6)	Ref
Season								
Influenza	2132	2003	94.0 (92.9–94.9)	1.5 (1.0–2.3); *P* = 0.04	220	67	30.5 (24.4–37.0)	0.7 (0.4–1.2); *P* = 0.19
Non-influenza	318	277	87.1 (82.9–90.6)	Ref	89	34	38.2 (28.1–49.1)	Ref
Hospital								
A	675	644	95.4 (93.5–96.9)	4.1 (2.0–8.5); *P* < 0.01	121	32	26.4 (18.8–35.2)	0.5 (0.2–1.4); *P* = 0.20
B	472	419	88.8 (85.6–91.5)	1.6 (0.8–3.3); *P* = 0.16	94	29	30.9 (21.7–41.2)	0.6 (0.2–1.5); *P* = 0.28
C	344	325	94.5 (91.5–96.6)	3.1 (1.4–6.6); *P* < 0.01	39	19	48.7 (32.4–65.2)	1.2 (0.5–3.2); P = 0.69
D	483	468	96.9 (94.9–98.3)	3.7 (1.5–9.3); *P* < 0.01	10	2	20.0 (2.5–55.6)	0.2 (0.03–1.8); *P* = 0.17
E	219	208	95.0 (91.2–97.5)	2.3 (0.8–6.3); *P* = 0.12	5	1	20.0 (0.5–71.6)	0.2 (0.02–3.6); *P* = 0.30
F	126	125	99.2 (95.6–99.9)	12.9 (1.6–106.7); *P* = 0.02	–	–	–	–
G	131	91	69.5 (60.8–77.2)	Ref	40	18	45.0 (29.3–61.5)	Ref
Setting where the test was ordered								
ED^c^	1492	1410	94.5 (93.2–95.6)	1.4 (0.9–2.0); *P* = 0.06	192	69	35.9 (29.2–43.2)	1.9 (1.1–3.5); *P* = 0.03
Inpatient	958	870	90.8 (88.9–92.6)	Ref	117	32	27.4 (19.5–36.4)	Ref

^a^Each analysis was adjusted for patient age, Charlson comorbidity index, year of admission, hospital length of stay and three other variables in this table

^b^111 patients who were ordered both rapid and multiplex PCR were included in this group for the purpose of this analysis

^c^This group included 22 patients who were ordered an influenza test both in ED and inpatient wards

For patients whose test results were received before discharge, the sensitivity varied across hospitals from 69.5% at Hospital G (the children’s hospital) to 99.2% at Hospital F. Most general hospitals (except hospitals B and E) had a significantly higher likelihood of having correct ICD-10-AM coding vs the children’s hospital (Hospital G)—e.g. Hospital F was 12.9 times more likely to have correct ICD-10-AM coding for influenza than Hospital G (OR, 12.9; 95% CI, 1.6–106.7; *P* = 0.02) after adjustment for potential confounders. There was also a significant variation in sensitivity by season of hospitalisation; hospitalisation during an influenza season was associated with significantly greater sensitivity (vs non-influenza season) (94.0 vs 87.1%; *P* = 0.04). However, for patients whose test results were received after discharge, there were no significant differences in sensitivity across hospitals or by season of hospitalisation.

Comparison of sensitivity by *setting where the test was ordered* did not reveal a significant difference for patients whose test results were received before discharge, although there was a trend toward significance (*P* = 0.06). For patients whose test results were received after discharge, however, ordering of a test in the ED was associated with a greater sensitivity than ordering in inpatient wards (35.9 vs 27.4%; *P* = 0.03) (Table 4).

## Discussion

### Key findings

This multicentre study evaluated the accuracy of ICD-10-AM coding for influenza in administrative data using laboratory results as a *gold standard*. The major finding is that the ICD-10-AM coding showed a moderately high sensitivity overall although there was considerable variation across study hospitals. The specificity, PPV and NPV were generally high. We found that sensitivity was substantially improved for patients whose test results were available before hospital discharge compared to those whose test results were received after discharge.

### Interpretation and comparison with existing literature

Whilst several studies have investigated the accuracy of ICD coding of other infectious diseases [11–16, 19–22], the few studies that have evaluated quality of influenza coding have been based in the US using ICD-9 [17, 18]. Studies by Feemster et al. [17] and Keren et al. [18] evaluated the accuracy of ICD-9 coding for influenza against results of laboratory tests (rapid test, PCR or viral culture) among a paediatric population. Feemster et al. conducted a multicentre study across three children’s hospital and reported a sensitivity of 72.5%. Keren and colleagues conducted their study at the Children's Hospital of Philadelphia and found a sensitivity of 65%. The sensitivity of ICD-10-AM coding in our children’s hospital (i.e. 69.5%, Table 4) was approximately similar to the sensitivity values reported in these studies despite differences in the ICD classification and laboratory tests utilised.

Studies that compared ICD coding with laboratory results in other infectious diseases reported considerable variation in coding accuracy rates with sensitivity ranging from 12 to 98% [13–16, 19, 21]. This variation could be due to differences in setting, population studied, coder training, ICD version assessed or because of the difference in the comparator or the reference standard used in the assessment of accuracy across studies [11–16, 20–24]. A systematic review and meta-analysis by Goto and colleagues evaluated the accuracy of ICD-9-CM and ICD10 codes for selected health-associated infections including *Clostridium difficile infection* and *methicillin-resistant Staphylococcus aureus* (MRSA) [21]. Compared to laboratory diagnosis, ICD codes for *Clostridium difficile infection* had a pooled sensitivity of 76% (data from seven studies) with a sensitivity value ranging from 36.2 to 98% across studies. For MRSA, two studies reported sensitivities of 24% and 59% [21].

In one Australian study, Das et al. evaluated the accuracy of ICD-10-AM codes for *Staphylococcus aureus* bacteremia (SAB) using a 10-year dataset from Canberra Hospital. They reported that, compared to results of blood culture, ICD-10-AM coding had a sensitivity of 55% for all cases of SAB and only 12% for a subset of patients with hospital-associated SAB [13]. However, separate analysis by test result availability was not conducted to determine if coding accuracy improved for patients whose test result were available before discharge. One reason for the difference in coding accuracy between this and our findings could potentially be the difference in laboratory tests used (blood culture *versus* PCR-based tests). Blood culture results take up to 5 days to return and are more likely to be pending at discharge [31, 32] which might have affected coding accuracy.

Laboratory testing plays a major role in ensuring accurate diagnostic coding for infectious diseases. Timely availability of test results while patients are in the hospital can therefore improve the coding accuracy given the result of a laboratory test can be reviewed by physicians during the episode of care and documented in the discharge summary. In this study, consistent with our original hypothesis, we observed a substantial improvement in coding accuracy among patients whose laboratory results were received before discharge with a sensitivity of 93.1% compared to only 32.5% for those receiving results after discharge. Our finding is consistent with a previous US study that assessed the accuracy of ICD-9 for *Clostridium difficile infection* [33]*.* That study reported an improvement in the sensitivity from 71% for all sample to 88% when the analysis was limited to patients whose test results were received before discharge [33]. Suboptimal coding accuracy observed among patients whose results were received after discharge was mainly due to a *missed diagnosis* (i.e. ICD10−/PCR+)—meaning that patients were not assigned influenza codes although test results eventually came back positive. This clearly suggests that timely availability of results during the clinical encounter could facilitate improved accuracy of ICD-10-AM coding. In this study, 13.1% of patients had test results received after discharge. It is important to remember that delay in processing results may not be controlled by health systems. However, health systems should have a mechanism in place to follow-up and document the results of laboratory tests to improve coding accuracy in the administrative database.

Interestingly, rapid PCR use and admission during an influenza season were associated with greater coding accuracy. Faster delivery of test results when rapid PCR was used might have resulted in timely and accurate documentation of the disease, although this requires further investigation. On the other hand, given that sensitivity and specificity values often vary with a *pre-test probability* of disease [34], the greater coding accuracy during the influenza season compared to the non-influenza season was likely due to higher *pre-test probability* of influenza during influenza season.

### Implications for practice and policy

Our findings have important implications both for clinical practice and health policy. In the current study, ICD-10-AM coding failed to identify 378 of test positive patients and miscoded 146 patients with negative test results. Missed and miscoded diagnoses are examples of diagnostic error and can have a direct impact on patient care quality and safety [35]. Patients with missed diagnoses may include a subgroup of patients for whom it was not recognised that they had influenza during their hospital stay. This may lead to missed opportunities for timely and appropriate treatment of the patient and precautions to prevent the potential spread of infection. Alternatively, patients with miscoded diagnoses might have been wrongly treated and unnecessarily consumed hospital resources. Further studies are needed to explore patients' experiences and to quantify the potential health and economic impacts of missed/miscoded diagnoses in infectious diseases in general.

Given that data generated through diagnostic coding are used in decision-making for reimbursement and resource allocation, inaccurate coding can lead to potential loss and unfair resource allocation [36] from a health policy perspective. In the current study, inaccurate coding was an issue particularly among patients with a pending test result at discharge with over two-thirds of these patients receiving inaccurate ICD-10-AM codes for influenza. This could be due to inadequate documentation of test-related information in the hospital discharge summaries [31] and poor test-result communication and follow-up. Our finding reinforces previous studies that have highlighted the importance of making laboratory results the main criteria for infectious disease ICD-10 coding to improve its accuracy [13, 15].

### Strengths and limitations

To our knowledge, this is the first study to evaluate the accuracy of ICD-10-AM coding for influenza against laboratory findings. Our study is a multicentre study that involved seven hospitals (six general and one children’s) enhancing the generalisability of results. The use of laboratory results as a *gold standard* is another strength. Molecular PCR-based assays used in this study are considered to be the best available methods to diagnose influenza [4, 29], providing objective measurements of the presence or absence of the disease.

Our study has several limitations. Although test result availability at discharge was the main factor affecting coding accuracy, a significant variation was observed across study hospitals, by type of PCR used and season of hospitalisation. Most general hospitals had significantly higher coding accuracy (sensitivity of > 90%) than the children’s hospital (sensitivity of < 70%). The current study did not investigate the potential reasons why such variation existed. Future research should explore the reasons for variation in coding accuracy across different types of hospitals including the potential role of organisational factors such as the work practices of clinical coders and differences in protocols of care across hospitals (including compliance with protocols) and physician-related factors, such as the clinician type and accuracy of clinical documentation [37]. Whilst we believe that laboratory results might have been used in the coding process especially for patients whose test results were available during the episode of care, the interpretation of our findings is limited by a lack of information on whether the test results were actually reviewed by physician, and decisions were made based on the results. Our study showed that of patients with pending test results at discharge, 14.3% returned positive for influenza suggesting potentially actionable results. However, we did not have access to data to assess any relevant follow-up actions (e.g. whether treatment decisions were made after discharge). Another limitation of this study is that unlike earlier studies [12, 14, 17], we did not conduct a subsequent medical record review to investigate patients with discordant findings between laboratory results and ICD-10-AM coding status (i.e. ICD10−/PCR+ or ICD10+/PCR−). Understanding the reasons for missing ICD-10-AM codes despite positive test results would have been valuable particularly for the subset of patients whose test results were received before discharge.

## Conclusion

The ICD-10-AM coding for influenza demonstrated high sensitivity, specificity, PPV and NPV against laboratory results when test results were available before hospital discharge. However, the accuracy of ICD-10-AM coding for influenza was substantially lower with a sensitivity of only 32.7% for patients whose test results were not available at the time of discharge. Findings indicate that the timely availability of laboratory results during the clinical encounter facilitates improved coding accuracy.

## Supplementary Information

**Additional file 1.** Table with ICD-10-AM codes for influenza by laboratory test results (n = 2527).

**Additional file 2.** Table with influenza diagnosis status vs laboratory test results across study hospitals.

## Data Availability

The data that support the findings of this study are available from the South Eastern Sydney Local Health District, but restrictions apply to the availability of these data, which were used under license for the current study, and so are not publicly available. Data are however available from the authors upon reasonable request and with permission of the South Eastern Sydney Local Health District.

## Abbreviations

APDC: Admitted patient data collection
CI: Confidence interval
ED: Emergency department
ICD-10-AM: International classification of diseases version 10-Australian modification
IQR: Inter-quartile range
LIS: Laboratory information system
MRSA: Methicillin-resistant *Staphylococcus aureus*
NPV: Negative predictive value
PCR: Polymerase chain reaction
PPV: Positive predictive value
RP: Respiratory panel
RSV: Respiratory syncytial virus
SAB: *Staphylococcus aureus* Bacteremia
