# Supplementary material for: Evaluation of the accuracy of diagnostic coding for influenza compared to laboratory results: the availability of test results before hospital discharge facilitates improved coding accuracy
Source: BMC Med Inform Decis Mak. 2021 May 22;21:168. doi: 10.1186/s12911-021-01531-9 (PMC8141245; doi:10.1186/s12911-021-01531-9)
Supplement: Supplementary file 1 — Additional file 1. Table with ICD-10-AM codes for influenza by laboratory test results (n = 2527). [file 12911_2021_1531_MOESM1_ESM.docx]

**Additional file 1**: Table with ICD-10-AM codes for influenza by laboratory test results (n=2,527).

| ICD-10-AM J09-J11 | Laboratory results | | |
| --- | --- | --- | --- |
|  | Positive | Negative | All (%) |
| J10.1: Influenza with other respiratory manifestations, seasonal influenza virus identified | 1485 | 72 | 1557 (61.6) |
| J10.0: Influenza with pneumonia, seasonal influenza virus identified | 697 | 29 | 726 (28.7) |
| J10.8: Influenza with other manifestations, seasonal influenza virus identified | 121 | 7 | 128 (5.1) |
| J09: Influenza due to identified zoonotic or pandemic influenza virus | 49 | 3 | 52 (2.1) |
| J11.1: Influenza with other respiratory manifestations, virus not identified | 21 | 26 | 47 (1.9) |
| J11.0: Influenza with pneumonia, virus not identified | 6 | 5 | 11 (0.4) |
| J11.8: Influenza with other manifestations, virus not identified | 2 | 4 | 6 (0.2) |
| Total | 2381 (100.0) | 146 (100.0) | 2527 (100.0) |
