# Supplementary material for: Evaluation of the accuracy of diagnostic coding for influenza compared to laboratory results: the availability of test results before hospital discharge facilitates improved coding accuracy
Source: BMC Med Inform Decis Mak. 2021 May 22;21:168. doi: 10.1186/s12911-021-01531-9 (PMC8141245; doi:10.1186/s12911-021-01531-9)
Supplement: Supplementary file 2 — Additional file 2. Table with influenza diagnosis status vs laboratory test results across study hospitals. [file 12911_2021_1531_MOESM2_ESM.docx]

**Additional file 2**: Table with influenza diagnosis status vs laboratory test results across study hospitals.

| Hospitals | PCR+/  ICD10+ | PCR+/  ICD10– | PCR–/  ICD10+ | PCR–/  ICD10– | Sensitivity  (95% CI) | Specificity  (95% CI) | PPV  (95% CI) | NPV  (95% CI) |
| --- | --- | --- | --- | --- | --- | --- | --- | --- |
| A | 676 | 120 | 37 | 3312 | 84.9 (82.2-87.3) | 98.9 (98.5-99.2) | 94.8 (92.9-96.3) | 96.5 (95.8-97.1) |
| B | 448 | 118 | 51 | 2114 | 79.2 (75.6-82.4) | 97.6 (96.9-98.2) | 89.8 (86.8-92.3) | 94.7 (93.7-95.6) |
| C | 344 | 39 | 8 | 1660 | 89.8 (86.3-92.7) | 99.5 (99.1-99.8) | 97.7 (95.6-99.0) | 97.7 (96.9-98.4) |
| D | 470 | 23 | 17 | 2468 | 95.3 (93.1-97.0) | 99.3 (98.9-99.6) | 96.5 (94.5-98.0) | 99.1 (98.6-99.4) |
| E | 209 | 15 | 12 | 723 | 93.3 (89.2-96.2) | 98.4 (97.2-99.2) | 94.6 (90.7-97.2) | 98.0 (96.7-98.9) |
| F | 125 | 1 | 11 | 573 | 99.2 (95.7-100) | 98.1 (96.7-99.1) | 91.9 (86.0-95.9) | 99.8 (99.0-100) |
| G | 109 | 62 | 10 | 2684 | 63.7 (56.1-70.9) | 99.6 (99.3-99.8) | 91.6 (85.1-95.9) | 97.7 (97.1-98.3) |
| Overall | 2381 | 378 | 146 | 13534 | 86.3 (85.0-87.6) | 98.9 (98.7-99.1) | 94.3 (93.2-95.1) | 97.3 (97.0-97.5) |

PPV, Positive predictive value; Negative predictive value.
